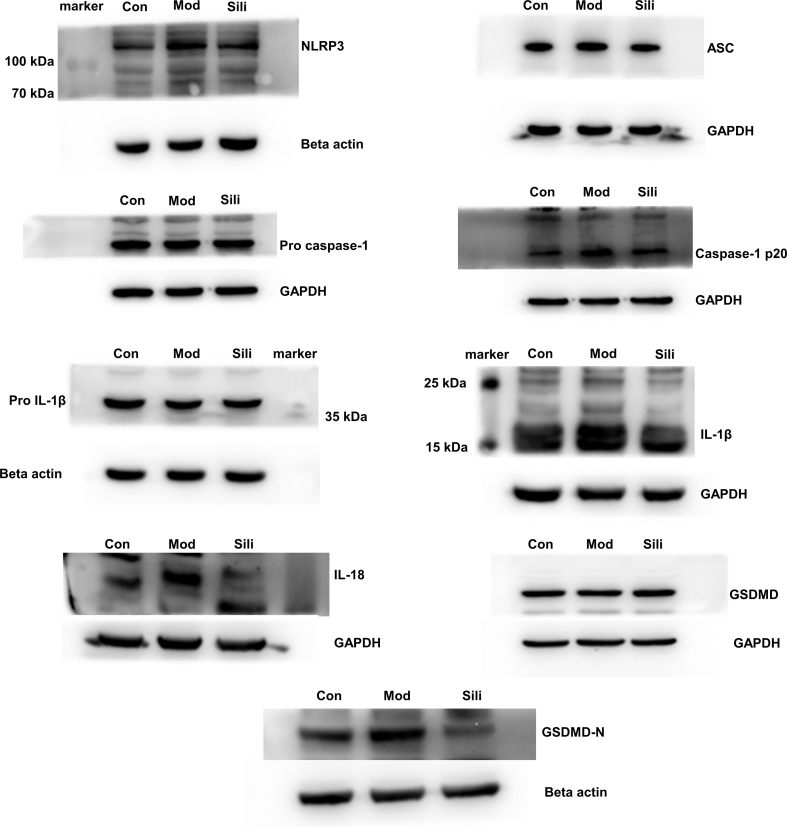# Silibinin ameliorates deoxycholic acid-induced pyroptosis in steatotic HepG2 cells by inhibiting NLRP3 inflammasome activation

**DOI:** 10.1016/j.bbrep.2023.101545

**Published:** 2023-09-13

**Authors:** Meiqing Mai, Ya Wang, Mengliu Luo, Zhongxia Li, Di Wang, Yongdui Ruan, Honghui Guo

**Affiliations:** a Department of Nutrition, School of Public Health, Guangdong Medical University, Dongguan, 523808, China; b BYHEALTH Institute of Nutrition & Health, Guangzhou, 510663, China; c Department of Traditional Chinese Medicine, The First Affiliated Hospital of Dongguan, Guangdong Medical University, Dongguan, 523710, China; d Dongguan Key Laboratory of Environmental Medicine, Guangdong Medical University, Dongguan, 523808, China

**Keywords:** Silibinin, Pyroptosis, NLRP3 inflammasome, Deoxycholic acid, Inflammation, Nonalcoholic steatohepatitis

## Abstract

Nonalcoholic steatohepatitis (NASH) represents an inflammatory subtype of nonalcoholic fatty liver disease (NAFLD). The activation of the NOD-like receptor protein 3 (NLRP3) inflammasome triggers pyroptosis, thus propelling the progression from simple steatosis to NASH. Silibinin, a hepatoprotective compound derived from milk thistle, exerts diverse hepatoprotective effects. However, the direct impact of silibinin on NLRP3 inflammasome activation and its ability to mitigate pyroptosis remain uncertain. To address this, we utilized an *in vitro* model of NASH, employing HepG2 cells treated with deoxycholic acid (DCA) and free fatty acids. Subsequently, we treated these model cells with silibinin for 24 h. Our findings demonstrated that, although there were no significant changes in cellular lipid content, silibinin effectively ameliorated hepatocyte injuries. Silibinin treatment inhibited the activation of the NLRP3 inflammasome and suppressed DCA-induced pyroptosis. Additionally, molecular docking analysis revealed that silibinin exhibited a binding affinity to components of the NLRP3 inflammasome similar to that of MCC950, a selective NLRP3 inhibitor. These results suggest that silibinin may alleviate inflammation in DCA-exposed HepG2 cells by mitigating pyroptosis, possibly through its binding affinity and inhibition of the NLRP3 inflammasome. Overall, our study indicates that silibinin holds promise as a therapeutic agent for NASH by modulating pyroptosis and inhibiting NLRP3 inflammasome activation.

## Highlights

• Silibinin mitigates inflammation and injury in steatotic hepatocytes.
• Silibinin inhibits activation of the NLRP3 inflammasome induced by deoxycholic acid.
• Silibinin improves deoxycholic acid-induced pyroptosis in steatotic hepatocytes.
• Silibinin exhibits the potential for direct interaction with the NLRP3 inflammasome.

## Graphical abstract

Silibinin shows promise in directly inhibiting the activation of the NLRP3 inflammasome and preventing the translocation of the N-terminal domain of gasdermin D to the cell membrane. This blockade effectively prevents membrane perforation and the subsequent release of mature interleukin-1 beta (IL-1β) and IL-18, thereby attenuating cellular injury and pyroptosis in HepG2 cells stimulated with free fatty acids and deoxycholic acid.

**Figure:**
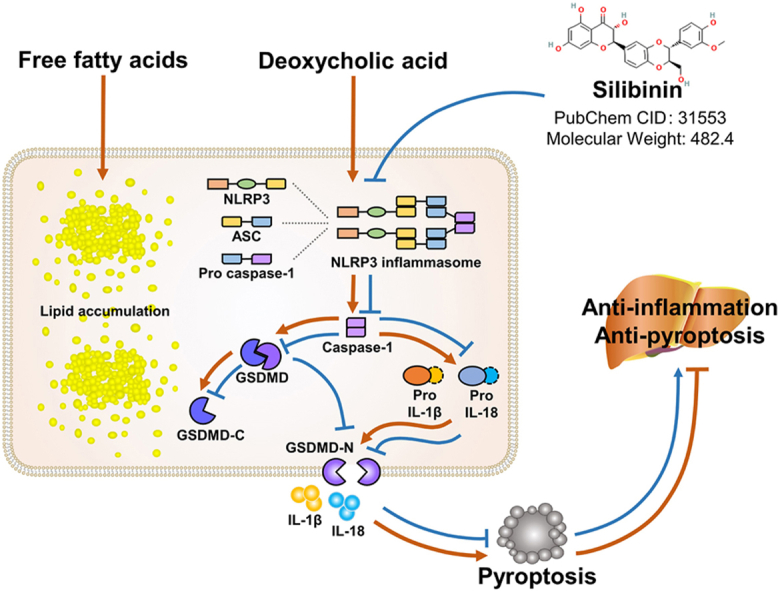

## Abbreviations

ALT: alanine aminotransferase
ASC: apoptosis-associated speck-like protein containing a caspase recruitment domain
BAs: bile acids
DCA: deoxycholic acid
HCC: hepatocellular carcinoma
GSDMD: gasdermin D
IL-1β: interleukin-1 beta
NAFLD: nonalcoholic fatty liver disease
NASH: nonalcoholic steatohepatitis
NLRP3: NOD-like receptor protein 3
OA: sodium oleate
PA: sodium palmitate
TC: total cholesterol
TG: triglyceride

## 1 Introduction

Nonalcoholic fatty liver disease (NAFLD) is a prevalent liver disorder worldwide. Within its broad spectrum, nonalcoholic steatohepatitis (NASH) represents the inflammatory subtype characterized by steatosis, hepatocyte injury (ballooning), and inflammation, with or without fibrosis [1]. The “two-hits” hypothesis, proposed in 1998, suggests that the progression from simple steatosis (early stage NAFLD) to NASH involves a second hit comprising inflammation, oxidative stress, lipid peroxidation, and mitochondrial dysfunction, following the initial steatosis, which serves as the first hit [2]. A recent meta-analysis estimated that approximately 24% of the global population is affected by NAFLD [3]. Moreover, NASH can lead to liver cirrhosis, significantly increasing the risk of hepatocellular carcinoma (HCC). While the annual incidence of HCC in patients with simple fatty liver is 0.44 per 1000 person-years, it surges to 5.29 per 1000 person-years, which is 12 times higher in individuals with NASH [3]. Early identification and effective management to halt or reverse NASH are crucial for improving the long-term prognosis of NAFLD.

Pyroptosis, a form of programmed cell death discovered by Zychlinsky in 1992 [4] and named in 2000, has gained significant attention in recent years. Initially believed to be solely involved in the innate immune response against intracellular bacteria [5], accumulating evidence indicates that pyroptosis also plays a crucial role in sterile inflammation, including chronic liver diseases [6,7]. This process involves the activation of nucleotide-binding oligomerization domain (NOD)-like receptors (NLRP3, NLRP1, NLRC4, NLRP9, and NLRP6) by pathogen-related molecular patterns (PAMPs) or damage-related molecular patterns (DAMPs) within the cytoplasm, thereby triggering caspase-1 activation and ultimately leading to pyroptosis [8].

The NOD-like receptor protein 3 (NLRP3) inflammasome has been demonstrated to play a pivotal role in the progression of NASH [9,10], a condition with a complex pathogenesis. Upon stimulation, NLRP3, identified as a key member of the NLR family, forms a large intracellular multiprotein complex with apoptosis-associated speck-like protein containing a caspase recruitment domain (ASC), activating caspase-1 and resulting in the proteolytic activation of interleukin-1 beta (IL-1β) and IL-18 [11]. Caspase-1 also cleaves gasdermin D (GSDMD) to relieve autoinhibition of its N-terminal domain (GSDMD-N), which exhibits increased levels in human and experimental NASH [12]. GSDMD-N perforates cell membranes, promoting the secretion of IL-1β and IL-18, triggering pyroptosis, and subsequently initiating an inflammatory cascade [13]. Mridha et al. found that MCC950, a selective NLRP3 inhibitor, could improve inflammation in mice fed a methionine/choline-deficient (MCD) diet [14]. Targeting the NLRP3 inflammasome and the resulting pyroptosis shows promise for the treatment of NASH [15].

Bile acids (BAs) are amphipathic molecules synthesized exclusively in the liver from cholesterol. They are released into the gastrointestinal tract to facilitate the absorption of dietary fats, steroids, vitamins, and drugs [16]. However, hydrophobic BAs, such as deoxycholic acid (DCA) and lithocholic acid (LCA), are known to exhibit significant cytotoxicity [17]. In individuals with NASH, as opposed to simple steatosis, higher levels of two distinct types of keto-bile acids, namely 7-keto-DCA and 7-keto-LCA, have been observed [18]. Belgaumkar et al. conducted a study comparing the effect of laparoscopic sleeve gastrectomy on BA profiles in pre-operative and post-operative NASH patients. The study found a significant reduction in plasma DCA levels after the surgery, along with a decrease in inflammatory cytokines and liver injury markers [19]. In our previous study, we confirmed that DCA could induce inflammation in steatotic hepatocytes by inhibiting PINK1-mediated mitophagy and activating the NLRP3 inflammasome [20]. Furthermore, the serum BA profiles of NASH patients were similar to those with other chronic liver diseases, with higher DCA levels observed in hepatitis B and C, compared to alcohol-induced liver disease and primary biliary cirrhosis [21]. Therefore, to establish a cell model of steatotic and inflammatory hepatocytes, we employed a combination of free fatty acids and DCA.

NASH is a complex condition with variable coexisting metabolic complications, which makes its treatment challenging. Although there are currently no specific pharmaceuticals approved by the US Food and Drug Administration (FDA) for NASH, certain drugs and chemicals have shown promise in randomized trials [1,22]. However, the intermediate metabolites of these drugs may cause toxic damage to hepatocytes and adverse reactions in other diseases [23,24]. Consequently, natural bioactive components have gained attention as potential preventive and therapeutic options for NASH.

Silymarin is a mixture of flavonoids extracted from milk thistle (*Silybum marianum* (L.) Gaertn) seeds, with silibinin being the major hepatoprotective compound, constituting 50%–60% of silymarin [25]. Chemically, silibinin is known as (2R,3R)-3,5,7-trihydroxy-2-[(2R,3R)-3-(4-hydroxy-3-methoxyphenyl)-2-(hydroxymethyl)-2,3-dihydro-1,4-benzodioxin-6-yl]-2, 3-dihydrochromen-4-one. It possesses a molecular formula of C_25_H_22_O_10_ and a molecular weight of 482.441. Silibinin's robust anti-inflammatory and antioxidant properties are attributed to its chemical structure, which features five hydroxyl (OH) groups, with three of them (5-OH, 7-OH and 20-OH) displaying phenolic characteristics. Silibinin exhibits various biological effects, including antioxidation, anti-inflammation, anti-fibrosis, and regulation of insulin resistance [26]. It has shown particular efficacy in treating chronic hepatitis due to its anti-inflammatory properties [27]. In addition to its inhibitory effects on viral hepatitis [28,29] and alcoholic liver injury [30], numerous studies have demonstrated the effectiveness of silibinin in improving NASH by reducing inflammation [[31], [32], [33]].

The objective of this study was to investigate the potential anti-inflammatory effect of silibinin against DCA-induced pyroptosis *in vitro* using HepG2 cells. Due to its small molecular size, silibinin is believed to readily permeate the cell membrane and exert a direct influence on the NLRP3 inflammasome. Our focus was on examining the binding affinity between silibinin and the NLRP3 inflammasome.

## 2 Materials and methods

### 2.1 Regents

Alanine amiotransferase (ALT), triglyceride (TG) and total cholesterol (TC) assay kits were purchased from Nanjing Jiancheng Biotechnology (Nanjing, Jiangsu, China). RNAios Plus, Pri-meScript™ RT reagent kit, and TB Green® Pre-mix Ex Taq™ II were obtained from Takara Biotechnology (Tokyo, Japan). The Caspase-1 Activity Assay Kit and Hoechst 33342/Propidium Iodide (PI) Double Staining Kit were purchased from Solarbio Science & Technology Co., Ltd. (Beijing, China). All other chemicals used were of analytical grade and commercially available.

The following primary antibodies were used: mouse anti-GAPDH (6C5) antibody from Beyotime (Shanghai, China), mouse anti-ASC/TMS1/PYCARD (F-9) antibody from Santa Cruz (Santa Cruz, CA), mouse anti-beta-actin (2D4H5) antibody from Proteintech (Wuhan, China), rabbit polyclonal antibody against GSDMD from Proteintech (Wuhan, China), and rabbit anti-cleaved N-terminal GSDMD (EPR20829-408) antibody from Abcam (Cambridge, UK). The rabbit polyclonal antibodies against NLRP3, pro caspase-1, caspase-1 p20, pro IL-1β, IL-1β, and IL-18 were obtained from Wanleibio (Shenyang, Liaoning, China).

### 2.2 Cell culture

The human hepatoma cell line HepG2 was obtained from the Cell Institute of the Chinese Academy of Sciences (Shanghai, China). HepG2 cells were cultured in Dulbecco's Modified Eagle Medium (DMEM) supplemented with 10% fetal bovine serum and 1% penicillin-streptomycin solution at 37 °C in a humidified atmosphere with 5% CO_2_. To model steatotic and inflammatory hepatocytes, we used sodium oleate (OA), sodium palmitate (PA), and deoxycholic acid (DCA). OA (Aladdin, Shanghai, China) and PA (Aladdin) were dissolved in a solution of fatty acid-free bovine serum albumin (BSA) at concentrations of 600 μM and 300 μM, respectively. DCA (Sigma-Aldrich, Shanghai, China) and silibinin (Macklin, Shanghai, China) were dissolved in dimethyl sulfoxide (DMSO) at concentrations of 800 mM and 100 mM, respectively. Stock solutions of DCA and silibinin were further diluted to 800 μM and 2 mM, respectively, using the culture medium. The final concentration of DMSO in all treatments was ≤0.1%.

HepG2 cells were divided into three groups: Group 1: Cells were incubated with DMEM containing an identical concentration of fatty acid-free BSA (control group, Con); Group 2: Cells were pretreated with 600 μM OA (dissolved in 20% fatty acid-free BSA), 300 μM PA (dissolved in 40% fatty acid-free BSA), and DCA for 24 h (model group, Mod); Group 3: Cells were pretreated with OA, PA and DCA at 37 °C for 24 h, and then treated with silibinin (dissolved in DMSO) at 37 °C for 24 h (silibinin group, Sili).

### 2.3 Cell viability assay

Cell viability was evaluated using the Cell Counting Kit-8 (CCK-8) [34]. Cells were seeded at a density of 5 × 10^3^/well in 100 μL of medium in 96-well microplates. After 24 h of various treatment, 10 μL of CCK-8 reagent was added to each well and incubated at 37 °C for 1 h. The absorbance was measured at 450 nm using a microplate reader (Infinite E Plex, Tecan, Shanghai, China), with wells without cells serving as blanks. The calculation formula for cell viability was as follows: Cell viability (%) = (A_450_, sample - A_450_, blank)/(A_450_, control - A_450_, blank) × 100%.

### 2.4 Oil red O staining

The cells were washed with phosphate-buffered solution (PBS) and fixed in 4% paraformaldehyde for 10 min. After fixation, the cells were briefly rinsed in 60% isopropanol and incubated with Oil Red O reagent for 10 min. Subsequently, the stained cells were washed in 60% isopropanol and counter-stained with hematoxylin for 1 min. After washing with distilled water, a drop of glycerol jelly mounting medium was added to the cells before they were observed under a microscope [35].

### 2.5 Measurement of caspase-1 activity, ALT, TG, and TC levels

Caspase-1 activity, ALT, TG, TC, and total protein levels were measured using commercially available kits following the manufacturer's protocols [30]. Absorbance readings were taken at specific wavelengths for each measurement. ALT levels were determined using the standard curve formula based on absorbance values. Caspase-1 activity, TG, and TC content were normalized to the total protein concentration.

### 2.6 Quantitative real-time PCR analysis

Total RNA was extracted from HepG2 cells using RNAios Plus. The concentration of RNA was determined by measuring the absorbance at 260 nm. Subsequently, cDNA was synthesized using the PrimeScript™ RT reagent kit. Samples were prepared using TB Green® Pre-mix Ex Taq™ II, and quantitative real-time PCR was conducted on a real-time Thermal cycler 5100 (Thermo Fisher Scientific, CA, USA). The relative concentrations of mRNA were normalized to the expression levels of GAPDH to quantify gene expression. Data analysis was performed using the 2^−△△CT^ method [36]. The primer sequences utilized in the PCR are provided in Supplementary Table S1.

### 2.7 Western blot analysis

The collected cells were lysed and homogenized using RIPA lysis buffer containing 1 mM phenylmethylsulfonyl fluoride. The protein content in the cell lysates was quantified using the BCA method. Approximately 20 μg of protein sample was subjected to sodium dodecyl sulfate polyacrylamide gel electrophoresis (SDS-PAGE) for separation and subsequently transferred to a PVDF membrane (Millipore, Tullagreen, IRL). Following blocking with 5% powdered skimmed milk, the PVDF membrane was incubated with primary antibodies overnight at 4 °C. After thorough washing with TBST three times, the PVDF membrane was incubated with an HRP-conjugated secondary antibody for 1 h on a shaker at room temperature [37]. Protein expression levels were assessed using Chemiluminescent HRP Substrate (Millipore), captured with FluorChem R (ProteinSimple, San Jose, CA), and analyzed using ImageJ 1.53c software (NIH, Bethesda, MD).

### 2.8 Immunofluorescence staining

HepG2 cells were fixed with 4% buffered paraformaldehyde for 20 min, permeabilized with 0.5% Triton-X for 10 min, and then blocked with 5% BSA for 1 h at room temperature. Subsequently, the cells were incubated with primary antibodies overnight at 4 °C, followed by treatment with coraLite488-conjugated or coraLite594-conjugated secondary antibodies for 1 h at room temperature. The nuclei were stained with DAPI or PI, and the results were captured using a TCS SP8 confocal microscope (Leica, Mannheim, Germany) [38].

### 2.9 Hoechst 33342/PI double staining

During the process of pyroptosis, the formation of pores in the cell membranes leads to the release of cellular contents and the staining of dead cells. This can be assessed using Hoechst 33342/PI double staining. Following the manufacturer's instructions, HepG2 cells from each group were stained with a mixture of 1 mL staining buffer, 5 μL Hoechst 33342, and 5 μL PI for 25 min at 4 °C [39]. Images were collected using an EVOS® FL Auto inverted fluorescence microscope (Thermo Fisher Scientific, CA, USA), and the percentage of PI-positive cells was calculated.

### 2.10 Molecular docking

AutoDock 4.2.6 software was employed to predict the binding affinity of the test compounds with the target proteins. The ligand structures were obtained from the PubChem website and adjusted to include hydrogens and charges. The 3D structures of NLRP3 (ID: 3QF2), ASC (2NK6), and caspase-1 (1ICE) proteins were downloaded from RCSB (https://www.rcsb.org/). Prior to docking, water molecules, salt ions, and small molecules were removed from the proteins. Polar hydrogens and charges were then added. Default docking parameters based on the Lamarckian Genetic Algorithm principle were utilized, with 50 runs performed. The docking procedure was subsequently executed. AutodockTools was employed to assess the docking results [40]. A molecular docking score above 6.5 indicated a strong binding between the target proteins and the small molecule silibinin. Additionally, the Root-Mean Square Deviation (RMSD) was calculated, with values below 2.0 Å considered favorable [41].

### 2.11 Statistical analysis

The experiments were conducted at least three times, and the results are presented as mean ± standard deviation (SD). Data analysis was performed using GraphPad Prism software (Version 8, San Diego, CA, USA). One-way ANOVA with multiple comparison post hoc analysis was employed to compare the mean values among groups. Statistical significance was defined as *p* < 0.05.

## 3 Results

### 3.1 Silibinin dose-dependently ameliorates DCA-induced inflammation and injury in steatotic hepatocytes

**Fig. 1:**
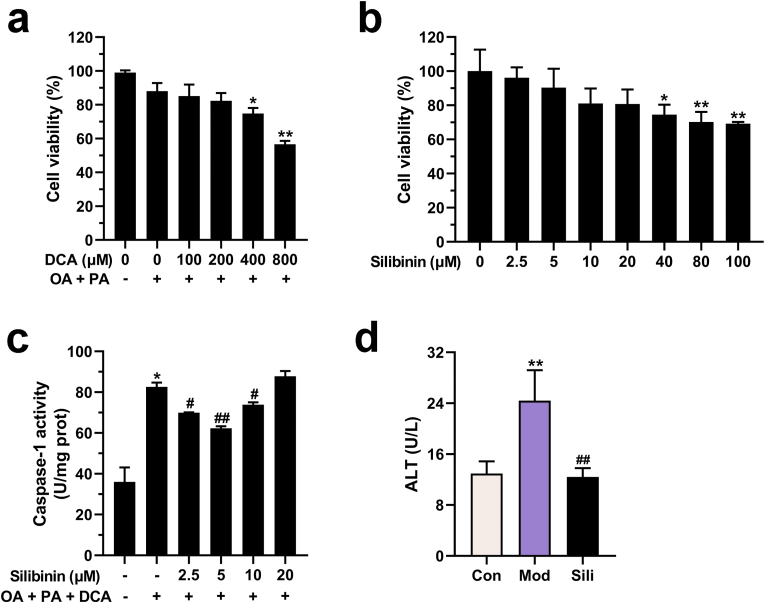
Silibinin mitigates hepatocellular inflammation and injury in HepG2 cells caused by DCA. **a**) HepG2 cells were treated with 600 μM OA, 300 μM PA, and various concentrations of DCA (0, 100, 200, 400, or 800 μM) for 24 h. Cell viability was determined using CCK-8 assay. **b**) Viability of HepG2 cells treated with different doses of silibinin (0, 2.5, 5, 10, 20, 40, 80, or 100 μM) for 24 h. **c-d**) Levels of caspase-1 activity and alanine aminotransferase (ALT). *p < 0.05 vs. control (Con), **p < 0.01 vs. Con, ^#^*p* < 0.05 vs. model (Mod), ^##^*p* < 0.01 vs. Mod. The graph bars depict the mean ± SD. All assays were performed in triplicate. Mod: Cells were subjected to pretreatment with 600 μM OA, 300 μM PA and 200 μM DCA. Sili: Cells were treated with 5 μM silibinin subsequent to modeling.

HepG2 cells were utilized to investigate the cytotoxicity of DCA and silibinin. The results obtained from the CCK-8 assay demonstrated that DCA induced cytotoxic effects on HepG2 cells when combined with OA and PA at concentrations of 400 and 800 μM, as shown in Fig. 1a. Subsequently, a concentration of 200 μM DCA was selected to induce inflammation in steatotic HepG2 cells, establishing an *in vitro* model of NASH. The anti-inflammatory effects of silibinin treatment on HepG2 cells were evaluated by assessing caspase-1 activity. The findings revealed that silibinin at concentrations of 2.5, 5, and 10 μM did not exhibit significant cytotoxicity in HepG2 cells, as depicted in Fig. 1b. However, it effectively attenuated DCA-induced caspase-1 activity in steatotic HepG2 cells after a 24-h period, as shown in Fig. 1c. Among these concentrations, 5 μM of silibinin demonstrated the most potent effect and was therefore chosen for subsequent experiments. Following the establishment of the model, the level of ALT in the culture supernatant increased, indicating cellular damage. However, treatment with silibinin significantly reduced the leakage of ALT, as illustrated in Fig. 1d. These findings suggest that silibinin has the potential to mitigate cell inflammation and injury caused by DCA in steatotic HepG2 cells.

### 3.2 Silibinin does not significantly influence lipid accumulation caused by free fatty acids

**Fig. 2:**
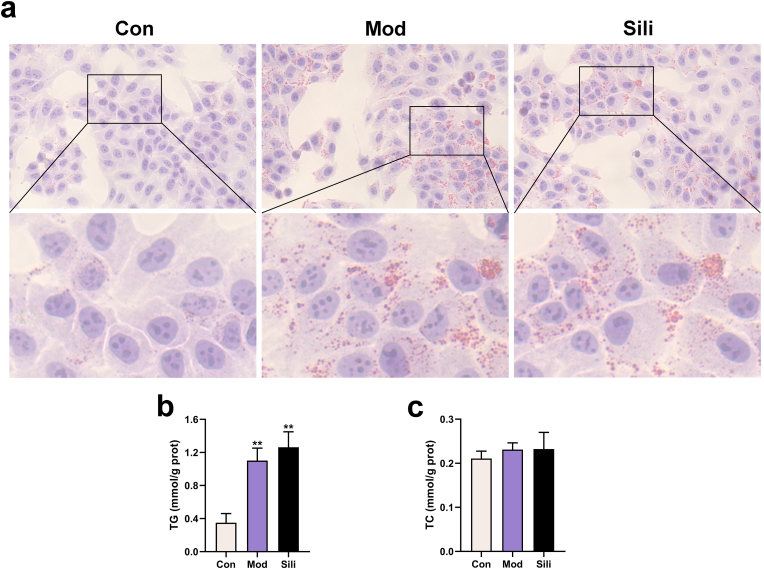
Silibinin does not significantly influence lipid accumulation caused by free fatty acids. **a**) Oil Red O staining of HepG2 cells (magnification: 400 × ). **b-c**) Intracellular TG and TC contents. *p < 0.05 vs. Con, **p < 0.01 vs. Con, ^#^*p* < 0.05 vs. Mod, ^##^*p* < 0.01 vs. Mod. The graph bars depict the mean ± SD. All assays were performed in triplicate. Mod: Cells were subjected to pretreatment with 600 μM OA, 300 μM PA and 200 μM DCA. Sili: Cells were treated with 5 μM silibinin subsequent to modeling. (For interpretation of the references to colour in this figure legend, the reader is referred to the Web version of this article.)

To assess the impact of silibinin on lipid accumulation, we employed Oil Red O staining and commercial kits to measure levels of TG and TC. Following the establishment of the model, we observed the presence of lipid droplets and a notable increase in TG content in HepG2 cells. However, treatment with silibinin did not exhibit any significant effect on lipid accumulation (Fig. 2a–b). Furthermore, there were no significant differences in TC content among the three groups (Fig. 2c).

### 3.3 Silibinin inhibits NLRP3 inflammasome activation in DCA-treated steatotic hepatocytes

**Fig. 3:**
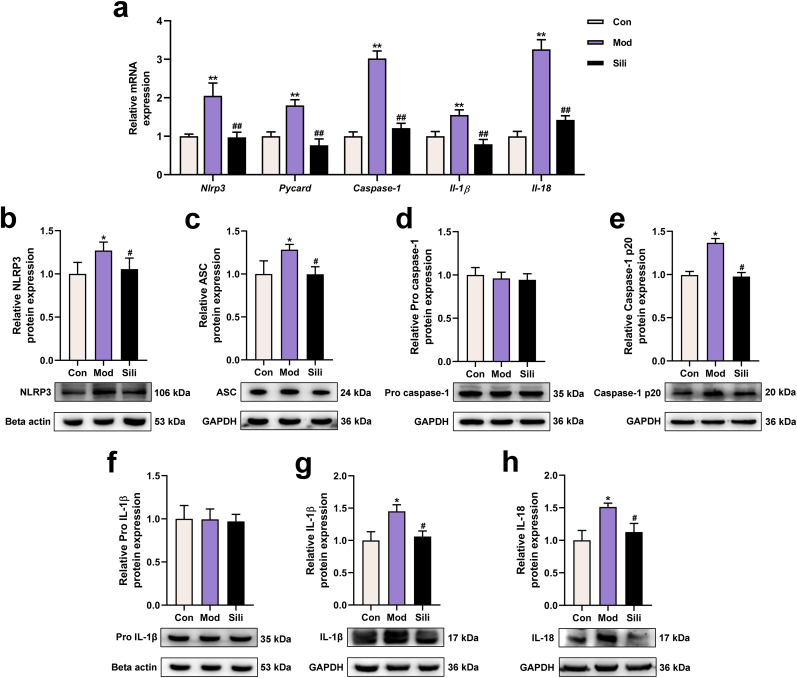
Silibinin downregulates the NLRP3 inflammasome signaling pathway to exert anti-inflammatory effects. **a**) mRNA levels of *Nlrp3*, *Pycard/Asc*, *caspase-1*, *Il-1β*, and *Il-18* in HepG2 cells were analyzed using qRT-PCR. **b-h**) Protein levels of NLRP3, ASC, pro caspase-1, caspase-1 p20, pro IL-1β, IL-1β, and IL-18 in HepG2 cells were determined using Western Blot analysis. GAPDH or beta-actin was used as the internal reference protein. *p < 0.05 vs. Con, **p < 0.01 vs. Con, ^#^*p* < 0.05 vs. Mod, ^##^*p* < 0.01 vs. Mod. The graph bars depict the mean ± SD. All assays were performed in triplicate. Mod: Cells were subjected to pretreatment with 600 μM OA, 300 μM PA and 200 μM DCA. Sili: Cells were treated with 5 μM silibinin subsequent to modeling.

We conducted further investigations to evaluate the impact of silibinin on NLRP3-mediated hepatocyte inflammation. As depicted in Fig. 3a, the qRT-PCR results demonstrated a significant downregulation of the transcription levels of *Nlrp3*, *Pycard/Asc*, *caspase-1*, *Il-1β*, and *Il-18* genes in the silibinin-treated group compared to the model group. The Western blot analysis revealed a substantial upregulation of NLRP3, ASC, caspase-1 p20, IL-1β, and IL-18 in the model group, which were significantly reduced in the presence of silibinin (Fig. 3b–c, e, and g-h). However, there was no observable difference in the precursors of caspase-1 p20 and IL-1β among the three groups (Fig. 3d, f). These findings suggest that silibinin exhibits the ability to inhibit the activation of the NLRP3 inflammasome.

### 3.4 Silibinin inhibits the translocation of GSDMD-N from the cytoplasm to the membrane in DCA-treated steatotic HepG2 cells

**Fig. 4:**
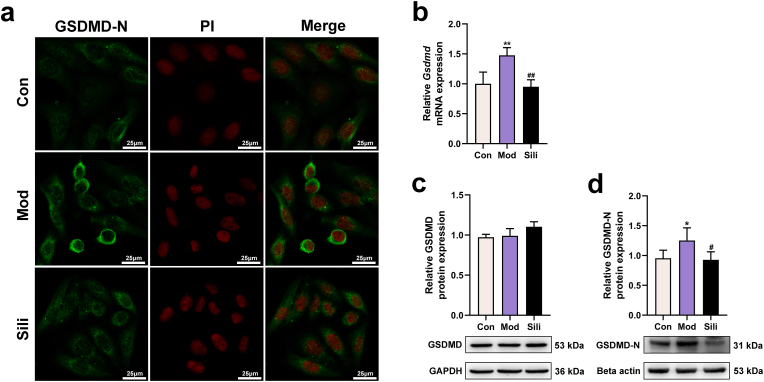
Silibinin inhibits the translocation of GSDMD-N from the cytoplasm to the membrane in DCA-treated steatotic HepG2 cells. **a**) Immunofluorescence assay showing the expression of GSDMD-N in HepG2 cells. The nuclei were stained with PI in red, and GSDMD-N was stained in green (magnification: 630 × ). Scale bar: 25 μm. **b**) mRNA levels of *Gsdmd* in HepG2 cells. **c-d**) Protein levels of GSDMD and GSDMD-N in HepG2 cells. *p < 0.05 vs. Con, **p < 0.01 vs. Con, ^#^*p* < 0.05 vs. Mod, ^##^*p* < 0.01 vs. Mod. The graph bars depict the mean ± SD. All assays were performed in triplicate. Mod: Cells were subjected to pretreatment with 600 μM OA, 300 μM PA and 200 μM DCA. Sili: Cells were treated with 5 μM silibinin subsequent to modeling. (For interpretation of the references to colour in this figure legend, the reader is referred to the Web version of this article.)

We conducted further investigations to evaluate the impact of NLRP3 inflammasome activation on GSDMD splicing, as the translocation of GSDMD-N from the cytoplasm to the membrane is a crucial event in inducing pyroptosis. Our findings revealed a significant increase in the translocation of GSDMD-N from the cytoplasm to the membrane in the model group following DCA treatment, as evidenced by enhanced immunofluorescence. However, treatment with silibinin notably suppressed this process (Fig. 4a). Additionally, we observed a decrease in the expression of the cleaved GSDMD-N pore-forming protein, while the expression of GSDMD itself remained unaltered, suggesting that silibinin exerts an anti-pyroptotic effect (Fig. 4b–d).

### 3.5 Silibinin suppresses DCA-induced NLRP3-ASC interaction

**Fig. 5:**
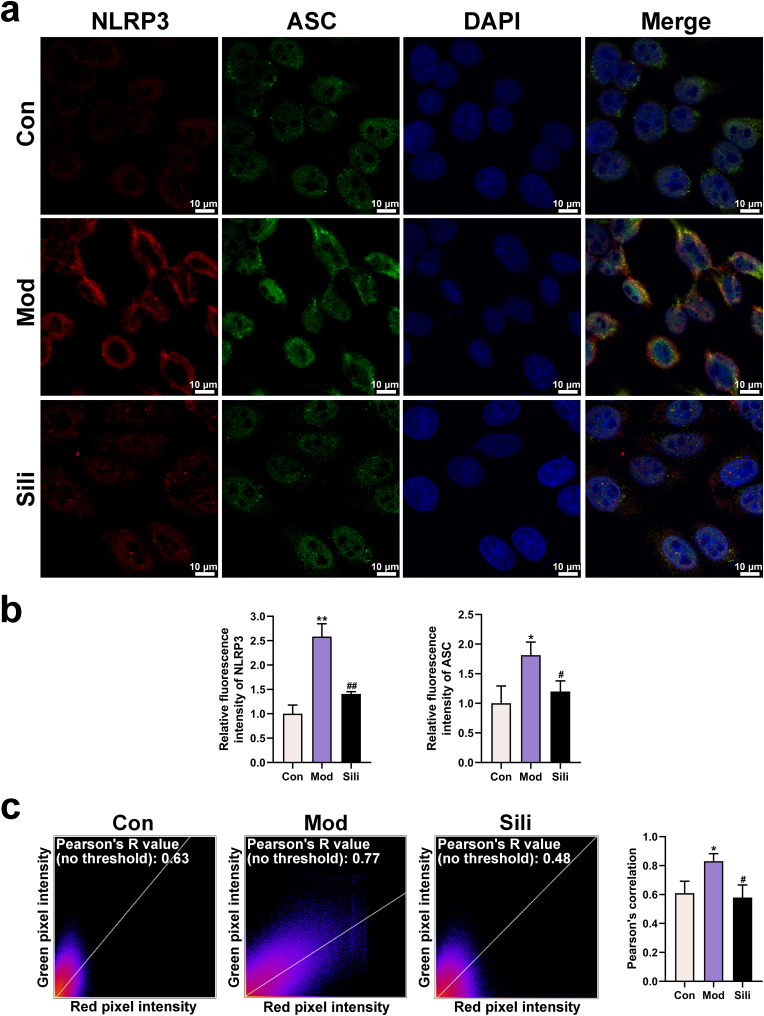
Silibinin interferes with NLRP3-ASC interaction. **a**) Representative images showing NLRP3-ASC interaction (magnification: 630 × , scale bar = 10 μm). The nuclei were stained with DAPI in blue, and targeted proteins were stained in green or red. **b**) Relative fluorescence intensity of NLRP3 and ASC in HepG2 cells. **c**) Quantitative analysis of NLRP3 and ASC colocalization. *p < 0.05 vs. Con, **p < 0.01 vs. Con, ^#^*p* < 0.05 vs. Mod, ^##^*p* < 0.01 vs. Mod. The graph bars depict the mean ± SD. All assays were performed in triplicate. Mod: Cells were subjected to pretreatment with 600 μM OA, 300 μM PA and 200 μM DCA. Sili: Cells were treated with 5 μM silibinin subsequent to modeling. (For interpretation of the references to colour in this figure legend, the reader is referred to the Web version of this article.)

The NLRP3-ASC interaction was visualized using a laser confocal microscope (Fig. 5a). We evaluated the protein levels of NLRP3 and ASC using immunofluorescence, and their interactions were determined through immunofluorescence colocalization. Compared to the control group, there was an increase in the expression of NLRP3 and ASC in HepG2 cells of the model group. Conversely, in the silibinin-treated group, the expression of NLRP3 and ASC was decreased (Fig. 5b), which was consistent with the qRT-PCR and Western blot results (Fig. 3a–c). By calculating Pearson's *R* value and the colocalization index with ImageJ software, we observed a significant induction of NLRP3-ASC colocalization in the cytoplasm due to DCA treatment, whereas silibinin exhibited the opposite effect (Fig. 5c). These findings suggest that silibinin inhibits the expression of NLRP3 and ASC, and suppresses the DCA-induced NLRP3-ASC interaction.

### 3.6 Silibinin inhibits DCA-induced pyroptosis in steatotic hepatocytes

**Fig. 6:**
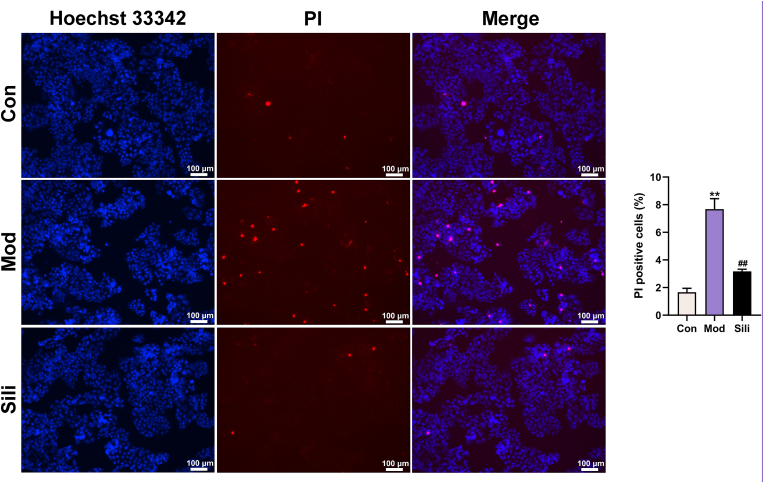
Silibinin reduces pyroptosis in steatotic and inflammatory HepG2 cells. Representative images of Hoechst 33342/PI staining. Scale bar is 100 μm. Proportions of PI staining areas were calculated. **p < 0.01 vs. Con, ^##^*p* < 0.01 vs. Mod. The graph bars depict the mean ± SD. All assays were performed in triplicate. Mod: Cells were subjected to pretreatment with 600 μM OA, 300 μM PA and 200 μM DCA. Sili: Cells were treated with 5 μM silibinin subsequent to modeling.

The Hoechst 33342 stain can permeate the entire cell membrane, whereas the PI stain cannot enter cells with intact membranes in normal or apoptotic states. However, it can pass through cell membranes via pyroptosis-related pores. Our findings demonstrated a significant increase in the proportion of PI staining in the model group compared to the control group. In contrast, the silibinin group showed a notable decrease in the proportion of PI staining compared to the model group, indicating a substantial inhibition of pyroptosis due to silibinin treatment (Fig. 6).

### 3.7 Silibinin exhibits binding affinity with the NLRP3 inflammasome

**Fig. 7:**
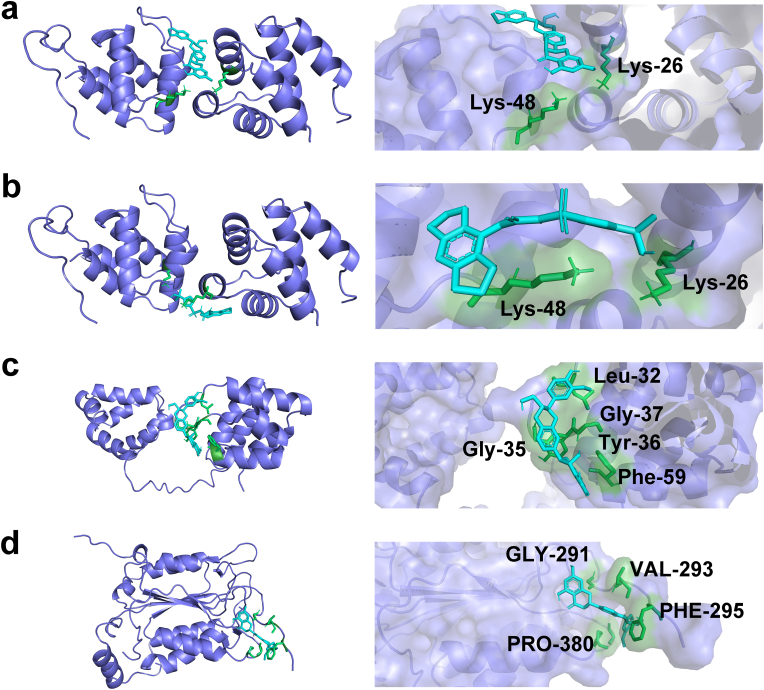
Docking pictures of test compounds and target proteins. **a**) NLRP3/silibinin. **b**) NLRP3/MCC950. **c**) ASC/silibinin. **d**) Caspase-1/silibinin.

Molecular docking analysis revealed a significant binding affinity between silibinin and the proteins NLRP3, ASC, and caspase-1. The docking scores obtained from the simulations are presented in Table 1, with scores exceeding 6.5, indicating a strong binding affinity between silibinin and the target proteins. Furthermore, the RMSD values, which were below 2.0 Å, provide additional evidence of favorable binding. To gain a more comprehensive understanding of the binding mode, the binding energy was evaluated. The combined free energies of NLRP3/silibinin, ASC/silibinin, and caspase-1/silibinin were determined to be −7.37 kcal/mol, −5.14 kcal/mol, and −5.35 kcal/mol, respectively. Notably, NLRP3/silibinin exhibited the highest binding energy, with a value of less than −7 kcal/mol. Additionally, the active site amino acids of NLRP3/silibinin were compared to the binding profile of MCC950, a selective inhibitor of NLRP3. The involvement of Lys-26 and Lys-48 in both silibinin and MCC950 suggests that silibinin has the potential to inhibit NLRP3 by binding to these similar active site amino acids (Fig. 7a–b). In the case of ASC, silibinin formed conventional hydrogen bonds with specific amino acids, including Leu-95, Gly-37, Tyr-36, Phe-59, and Gly-35 (Fig. 7c). When interacting with caspase-1, silibinin established hydrogen bonds with Gly-291, Val-293, Phe-295, Gly-382, and Pro-380 on the protein (Fig. 7d). The docking data strongly suggest that silibinin has the ability to directly target NLRP3 and its downstream signaling pathway, which was further confirmed by *in vitro* experiments.

**Table 1:** Docking of the test compounds with target proteins.

Target protein/test compound	Docking score	RMSD (Å)	Binding Energy (kcal/mol)
NLRP3/silibinin	−13.49	1.006	−7.37
ASC/silibinin	−11.273	0.088	−5.14
Caspase-1/silibinin	−11.44	0.336	−5.35
NLRP3/MCC950	−8.448	2.124	−6.08

## 4 Discussion

This study reveals the significant inhibitory effects of silibinin on the activation of NLRP3 inflammasome and the translocation of GSDMD-N in steatotic HepG2 cells induced by DCA. Activation of the NLRP3 inflammasome initiates the autoproteolytic activation of caspase-1, leading to the cleavage of GSDMD into GSDMD-N, which is then translocated to the cell membrane. Consequently, membrane perforation occurs, accompanied by the release of mature IL-1β and IL-18, both of which are cleaved by caspase-1, ultimately resulting in hepatocyte pyroptosis. Through molecular docking analysis, we have observed that silibinin directly binds to the NLRP3 inflammasome, effectively blocking this pathway and significantly reducing hepatocyte injury and inflammation.

Recent research has established a connection between gut dysbiosis and the progression of NAFLD, primarily mediated by the presence of metabolites, including BAs [42]. Studies have demonstrated the potential of specific BAs, such as tauroursodeoxycholic acid and ursodeoxycholic acid, in improving NAFLD [43,44]. However, in the case of NASH, certain species of BAs, including DCA, exert direct toxic effects on hepatocytes. Notably, DCA acts as a danger molecule by triggering NLRP3 inflammasome activation, inducing hepatocyte pyroptosis, and promoting the development of NASH [20,45]. Animal evidence suggests that blocking DCA production or modifying gut bacteria effectively prevents HCC development in obese mice [46]. Given that liver biopsy remains the preferred diagnostic method for NASH in humans [1], and establishing an animal model for NASH also presents significant challenges. Therefore, a more feasible approach may involve using free fatty acids and DCA to create a NASH cell model. Interestingly, elevated DCA levels have also been observed in certain chronic hepatitis cases [21], indicating that blocking the DCA-triggered signaling pathway could hold promise as a potential treatment strategy for these conditions.

Silymarin, a mixture containing silibinin, has been utilized for centuries and is increasingly recognized for its efficacy in promoting overall health, particularly in the treatment of liver diseases [47]. Silibinin has been found to alleviate hepatic inflammation caused by oxidative stress by upregulating Nrf2 target genes, inhibiting the release of proinflammatory mediators, and suppressing NF-κB signaling in mice fed a methionine/choline-deficient diet [48]. Acting as a partial agonist of peroxisome proliferator-activated receptor alpha (PPARα), a ligand-activated transcription factor associated with hepatic steatosis, silibinin protects against NAFLD in mice by reducing lipid accumulation. The lipid-lowering effect of silibinin can be attributed to the activation of PPARα [49]. Therefore, the primary mechanisms through which silibinin inhibits the progression of NAFLD involve enhancing antioxidant activity, suppressing inflammation, and improving lipid accumulation.

Our study has yielded intriguing findings regarding the effect of silibinin on hepatocyte lipid accumulation induced by free fatty acids in the presence of DCA intervention. In contrast to some *in vitro* experiments that commonly utilize free fatty acids to establish hepatocyte steatosis models [50,51], we observed that silibinin did not improve hepatocyte lipid accumulation when combined with DCA. In our previous study, we discovered that DCA, instead of exacerbating hepatocyte steatosis, induced hepatocyte inflammation by inhibiting mitophagy and promoting NLRP3 inflammasome activation [20]. However, NLRP3 inflammasome activation can also influence autophagy [52], leading to caspase-1-dependent mitophagy blockade in macrophages, resulting in dysfunctional mitochondria and an exacerbated inflammatory response [53]. Moreover, balanced autophagy can effectively mitigate lipid accumulation [54,55]. Therefore, we hypothesize that silibinin may selectively inhibit NLRP3 inflammasome activation to exhibit anti-inflammatory effects and alleviate hepatocyte injury in the presence of DCA-induced inflammation in hepatocytes.

A previous study on breast cancer demonstrated that silibinin treatment impairs mitochondrial dynamics, decreases ROS levels, and prevents NLRP3 inflammasome activation in MDA-MB-231 cells [56]. In the investigation of hepatic ischemia-reperfusion, silibinin has demonstrated a significant capacity to decrease serum ALT and AST activities while ameliorating inflammatory damage to liver tissue. This beneficial effect can be attributed to its capability to regulate the expression of NLRP3 genes [57]. Our study further demonstrates that silibinin can effectively reduce ALT levels in the culture supernatant and suppress the mRNA and protein expressions of NLRP3 inflammasome signaling targets in DCA-treated HepG2 cells, effectively preventing pyroptosis. Additionally, we propose that silibinin has the potential to pass through the cell membranes and directly interact with the NLRP3 inflammasome. This interaction likely inhibits the oligomerization of NLRP3, similar to MCC950 and NLRP3 [58], thereby impeding the assembly of the NLRP3 inflammasome, caspase-1 maturation, and downstream signaling. This hypothesis is supported by immunofluorescence localization and the simulation results of ligand-protein docking.

In conclusion, our findings highlight the ability of silibinin to alleviate inflammation in steatotic HepG2 cells exposed to DCA by ameliorating pyroptosis. The binding affinity and inhibition of the NLRP3 inflammasome may represent one of the underlying molecular mechanisms involved.

## Declarations

Funding: BYHEALTH Nutrition and Health Research Foundation (TY202101001); 10.13039/501100001809National Natural Science Foundation of China (82273622); Guangdong Basic and Applied Basic Research Foundation (2021B1515140057); Discipline Construction Project of Guangdong Medical University (4SG21016G).

## Availability of data and material

The analyzed data sets generated during the present study will be provided by the corresponding author on reasonable request.

## Authors' contributions

Meiqing Mai: Writing – original draft, Methodology, Formal analysis, Investigation. Ya Wang: Writing – original draft, Methodology, Formal analysis, Investigation. Mengliu Luo: Methodology, Investigation. Zhongxia Li: Methodology, Investigation. Di Wang: Writing – review & editing. Yongdui Ruan: Conceptualization, Resources, Writing – review. Honghui Guo: Conceptualization, Resources, Writing – review and editing, Project administration, Funding acquisition.

## Ethics approval and consent to participate

This paper only contains *in vitro* studies and raises no ethical concerns.

## Consent for publication

All authors have approved to submit the manuscript to your journal for publication.

## Declaration of competing interest

The authors declare that they have no known competing financial interests or personal relationships that could have appeared to influence the work reported in this paper.

## Figures and Tables

**Fig. s1:**