# Supplementary material for: Silibinin ameliorates deoxycholic acid-induced pyroptosis in steatotic HepG2 cells by inhibiting NLRP3 inflammasome activation
Source: Biochem Biophys Rep. 2023 Sep 13;35:101545. doi: 10.1016/j.bbrep.2023.101545 (PMC10507139; doi:10.1016/j.bbrep.2023.101545)
Supplement: Multimedia component 1 [file mmc1.docx]

**Silibinin** **ameliorates deoxycholic acid-induced** **pyroptosis in** **steatotic HepG2 cells by** **inhibiting NLRP3** **inflammasome activation**

Meiqing Mai ^a^, Ya Wang ^a^, Mengliu Luo ^a^, Zhongxia Li ^b^, Di Wang ^b^, Yongdui Ruan ^c^ and Honghui Guo ^a, d, *^

^a^ Department of Nutrition, School of Public Health, Guangdong Medical University, Dongguan 523808, China

^b^ BYHEALTH Institute of Nutrition & Health, Guangzhou 510663, China

^c^ Department of Traditional Chinese Medicine, the First Affiliated Hospital of Dongguan, Guangdong Medical University, Dongguan 523710, China

^d^ Dongguan Key Laboratory of Environmental Medicine, Guangdong Medical University, Dongguan 523808, China

^*^ To whom correspondence should be addressed at Department of Nutrition, School of Public Health, Guangdong Medical University, Dongguan 523808, China. E-mail: guohh1999@gdmu.edu.cn, ORCID ID: 0000-0001-7837-0392.

**Supplementary Table**

Table S1 Primers for qRT-PCR

| Gene | Forward primer 5' to 3' | Reverse primer 5' to 3' |
| --- | --- | --- |
| *Gapdh* | GGAGCGAGATCCCTCCAAAAT | GGCTGTTGTCATACTTCTCATGG |
| *Nlrp3* | GATCTTCGCTGCGATCAACAG | CGTGCATTATCTGAACCCCAC |
| *Pycard* | TGGATGCTCTGTACGGGAAG | CCAGGCTGGTGTGAAACTGAA |
| *Caspase-1* | TTTCCGCAAGGTTCGATTTTCA | GGCATCTGCGCTCTACCATC |
| *Gsdmd* | GTGTGTCAACCTGTCTATCAAGG | CATGGCATCGTAGAAGTGGAAG |
| *Il-1β* | ATGATGGCTTATTACAGTGGCAA | GTCGGAGATTCGTAGCTGGA |
| *Il-18* | TCTTCATTGACCAAGGAAATCGG | TCCGGGGTGCATTATCTCTAC |
